# Human circular RNA hsa_circ_0000231 clinical diagnostic effectiveness as a new tumor marker in gastric cancer

**DOI:** 10.1002/cnr2.2081

**Published:** 2024-05-04

**Authors:** Ronghua Fang, Wentao Yuan, Chunyan Mao, Jing Cao, Hongmei Chen, Xiuying Shi, Hui Cong

**Affiliations:** ^1^ Department of Laboratory Medicine Affiliated Hospital of Nantong University Nantong China; ^2^ Department of Clinical Medicine Medical School of Nantong University Nantong China; ^3^ Department of Blood Transfusion Affiliated Hospital of Nantong University Nantong China; ^4^ Vip Ward Affiliated Hospital of Nantong University Nantong China

**Keywords:** biomarkers, circular RNA, diagnostic, gastric cancer, hsa_circ_0000231

## Abstract

**Background:**

Owing to the subtlety of initial symptoms associated with gastric cancer (GC), the majority of patients are diagnosed at later stages. Given the absence of reliable diagnostic markers, it is imperative to identify novel markers that exhibit high sensitivity and specificity. Circular RNA, a non‐coding RNA, plays an important role in tumorigenesis and development and is well expressed in body fluids.

**Aims:**

In this study, we aimed to identify hsa_circ_0000231 as a new biomarker for the diagnosis of GC and to assess its clinical diagnostic value in serum.

**Methods and Results:**

The stability and correctness of hsa_circ_0000231 was determined by agarose gel electrophoresis, Rnase R assay and Sanger sequencing. Real‐time quantitative polymerase chain reaction (qRT‐PCR) was designed to discover the expression level of hsa_circ_0000231 and whether it has dynamic serum monitoring capability. The correlation between hsa_circ_0000231 and clinicopathological parameters was analyzed by collecting clinical and pathological data from GC patients. In addition, diagnostic efficacy was assessed by constructing receiver operating characteristic curves (ROC). Hsa_circ_0000231 exhibits a stable and consistently expressed structure. In GC serum, cells, and tissues, it demonstrates reduced expression levels. Elevated expression levels observed postoperatively suggest its potential for dynamic monitoring. Additionally its expression level correlates with TNM staging and neuro/vascular differentiation. The area under ROC curve (AUC) for hsa_circ_0000231 is 0.781, indicating its superior diagnostic value compared to CEA, CA19‐9, and CA72‐4. The combination of these four indicators enhances diagnostic accuracy, with an AUC of 0.833.

**Conclusions:**

The stable expression of hsa_circ_0000231 in the serum of gastric cancer patients holds promise as a novel biomarker for both the diagnosis and dynamic monitoring of GC.

## 1 INTRODUCTION

Gastric cancer (GC) is one of the most common malignant tumors in the world. The latest statistics need to show that the incidence and mortality of gastric cancer rank fifth (5.6%) and third (7.7%) in the world.^1^ GC is typically discovered at an advanced stage with malignant hyperplasia and lymph node metastases due to the lack of early signs.^2^ Upper gastrointestinal endoscopy and pathology biopsy remain the gold standard for clinical diagnosis, but the disadvantage is that they are overly intrusive.^3^ Since their introduction into the clinic, tumor markers including CEA, CA19‐9, and CA72‐4 have been widely employed as non‐invasive liquid biopsy procedures to provide assistance in diagnosis. However, given their low sensitivity and specificity, developing effective non‐invasive diagnostic markers for gastric cancer has become crucial for detection and therapy.^4^, ^5^ As a mean to find more diagnostic markers for cancer together with the well‐known TMs, lipocalins and adipokines were examined.^6^, ^7^, ^8^

Reverse splicing creates circular RNAs (CircRNAs), single‐stranded covalently closed endogenous RNA molecules that are mostly synthesized from precursor messenger RNAs lacking 5′ end caps or 3′ poly(A) tails.^9^, ^10^ In eukaryotes, where they are extensively expressed, circRNAs were first thought to be an editing mistake with no substantial biological consequences. Scientists have recently discovered and identified hundreds of circRNAs because of improvements in genomic and transcriptomic data produced by high‐throughput sequencing and bioinformatics tools.^11^ Studies have demonstrated that certain functionally described circRNAs serve important roles in gene regulation through a variety of activities, including functioning as translational functions or binding to RNA‐binding proteins as miRNA sponges.^12^, ^13^, ^14^ Most notably, circular RNA may regulate the expression of several pathways, including those involved in gastric cancer, liver cell carcinoma, lung cancer, colorectal cancer, to play a role in promoting or suppressing cancer during tumor growth.^15^, ^16^, ^17^, ^18^, ^19^ As a result, circular RNAs have the possibility of serving as therapeutic targets and biomarkers, opening promising opportunities for the early detection, monitoring, and prediction of tumors.^20^ For example, Ma et al. found that hsa_circ_0004872 inhibited GC growth, invasion, and metastasis in vitro and in vivo by acting as a miR‐224 “sponge” to upregulate the expression of p21 and Smad 4.^16^ Their results suggest that hsa_circ_0004872 may serve as a promising diagnostic and prognostic marker for gastric cancer patients. Consequently, it is reasonable to postulate that hsa_circ_0000231 may offer promising opportunities as a therapeutic target and biomarker for the early detection, monitoring, and prediction of tumors.

In this paper, we detected the expression level of hsa_circ_0000231 in cells, serum and tissues of GC patients and healthy physical examination subjects, and evaluated its diagnostic efficacy in GC and its correlation with clinicopathological parameters. The changes of serum hsa_circ_0000231 in GC patients before and after operation were analyzed.

## 2 MATERIALS AND METHODS

### 2.1 Clinical serum and tissue specimen collection

All clinical serum and tissue samples were available from Nantong University Hospital between November 2021 and August 2022. There were 96 patients with stomach cancer, 39 with gastritis, 26 with paired preoperative and postoperative gastric cancer patients, 74 with healthy serum samples, and 15 with paired gastric cancer and para cancerous tissue samples. In 1.5 mL RNase‐free EP tubes, the top serum was withdrawn and kept as a backup at −80°C. No chemotherapy or radiation was administered before the operation for any of the patients with gastric cancer in this trial who were determined to have primary gastric cancer by qualified pathologists and physicians.

### 2.2 Cell culture

Human gastric epithelial cells (GES‐1), together with human GC cell lines HGC‐27, MKN‐45, MGC‐803, and AGS, were purchased from the Stem Cell Bank of the Chinese Academy of Sciences in Shanghai, China. None of the cell lines described above was contaminated with mycoplasma. All cell lines were cultivated in RPMI1640 medium (Gibco, Grand Island, NY, #C11875500BT), complemented with 10% fetal bovine serum (FBS) (Procell, Wuhan, China, #164210–50), and 1% penicillin and streptomycin (NCM Biotech, Suzhou, China, #C100C8). The cells were maintained in a moist incubator at 37°C with 5% CO_2_.

### 2.3 RNA extraction

The Blood Total RNA Rapid Extraction Kit (BioTeke Corporation, Beijing, China, #RP1102) was utilized to extract total RNA from 300 μL of plasma and then used to extract RNA from serum. According to the recommendations, 600–800 mg of clinical tissue samples were extracted using TRIzol (Invitrogen, Germany, #15596018). One milliliter was lysed, extracted, and/or refrigerated at −80°C for storage. Cells under excellent growth conditions were either kept at −80°C in the refrigerator or digested with TRYSPIN 0.25% EDTA (Gibco, Grand Island, NY, #25200‐072) and lysed with TRIzol (Invitrogen, Germany, #15596018) 1 mL.

### 2.4 Real‐time quantitative polymerase chain reaction

The extracted total RNA was reverse transcribed to single‐stranded cDNA using a reverse transcription kit (Thermo Fisher Scientific, USA, #K1622) according to the instructions for the 20 μL system. Eleven microliter of total RNA, 4 μL of reaction buffer, 2 μL of 10 mM dNTP mix, 1 μL of random primer, 1 μL of RNase inhibitor, and 1 μL of RevertAid RT make up the kit. The combined solution was incubated in (BIO‐RAD, USA) for 60 min at 42°C and 5 min at 70°C. Real‐time quantitative polymerase chain reaction (qRT‐PCR) experiments were performed on the acquired cDNA using a Roche Light Cycler 480 (Roche, Switzerland). Each reaction system's total reaction volume was 20 μL, which included 10 μL of SYBR Green I Mix (ABclonal, Wuhan, Chiana, #RM21203), 6 μL of enzyme‐free water, 0.5 μL of primers, and 3 μL of cDNA. Ribo Biotechnology (Shanghai, China) manufactured the primers utilized in this investigation, and the target gene sequence used was hsa_circ_0000231: forward primer, 5′‐GCTCCACTGAACAGTTTTTA‐3′; reverse primer, 5′‐GGCTTGTTG GATGAATATAGCT, internal reference, 18s rRNA: reverse primer: 5′‐CCATCCAATCGGTAGTAGCG; forward primer: 5′‐GTAACCCGTTG AACCCAT. The relative expression of hsa_circ_0000231 was calculated by the 2^−ΔΔCT^ method, ΔΔCT = mean of the experimental group (CT_hsa_circ_0000231_ − CT_18SrRNA_) and control group (CT_hsa_circ_0000231_ − CT_18SrRNA_).

### 2.5 Agarose gel electrophoresis

1.6 g of agarose (Biosharp, Hefei, China, #BS081‐100g) was dissolved in 80 mL of 50× TAE (Biosharp, Hefei, China, #BL533A) solution and microwaved to dissolve. Allow it cool to around 50°C, then add 10 μL of Goldview nucleic acid dye (Biosharp, Hefei, China, #BS357A), shake, and mix well before gently pouring into the electrophoresis tank along the wall and inserting the comb immediately. After the gel has formed, remove the comb, place the gel plate in the electrophoresis tank, add the electrophoresis solution, add the proper quantity of PCR product and the loading buffer to the sample wells, and set the marker wells aside as the control wells. With a gel imager, the outcomes were seen after the electrophoresis conditions were adjusted at 110 V, 40 min (BIO‐RAD, USA).

### 2.6 Exonuclease digestion experiments

According to the RNA extraction procedure outlined above, RNA from MKN‐45 cells was extracted and divided into two equal portions. One portion of the RNA was treated with 3U Rnase R (20 U/μL, BIOSEARCH, CA, USA, #RNR07250) at 37°C for 30 min before being inactivated with Rnase R enzyme at 70°C, while the other portion was left untreated. Rnase R, cellular RNA, 10 μL buffer, and Rnase‐free water constituted a 20 μL system after reverse transcription to obtain cDNA and qRT‐PCR to detect its expression level.

### 2.7 Bioinformatics analysis

The basic information and characteristics of hsa_circ_0000231 is found in circBase database (http://www.circbase.org/). And we predicted the downstream miRNAs by CircBank database (http://www.circbank.cn/), Miranda (http://www.microrna.org/) and TargetScan database (http://targetscan.org/vert_80/), and searched the ENCORI database (https://rnasysu.com/encori/) for these miRNA expressions.

### 2.8 Statistical analysis

GraphPad Prism 9.0 (SanDiego, USA) was used to create the visuals, and SPSS 27.0 (IBM SPSS Statistics, Chicago, USA) was used to conduct statistical analyses of clinicopathological data. For the comparison of two independent samples, bilateral t test was used to ensure the accuracy and reliability of the results. For the comparison of multiple independent samples, ANOVA one‐way analysis of variance was used to explore the differences between groups more comprehensively. In addition, in order to study the relationship between serum hsa_circ_0000231 expression and clinical pathological parameters, Chi‐square test was used for statistical analysis. With *p* values <.05 considered as statistically significant, * means *p* < .05, ** indicates *p* < .01, *** indicates *p* < .001, ^****^ means *p* < .0001.

## 3 RESULTS

This study aims to examine the application of hsa_circ_0000231 in the diagnosis and assessment of the efficacy of GC patients. With low expression and an expression level that relate to therapy, we discovered via a series of in vitro tests that hsa_circ_0000231 is stable and reproducible in GC and has the potential to be employed as a biomarker for GC diagnosis and efficacy observation.

### 3.1 Basic information and characteristics of hsa_circ_0000231

**FIGURE 1:**
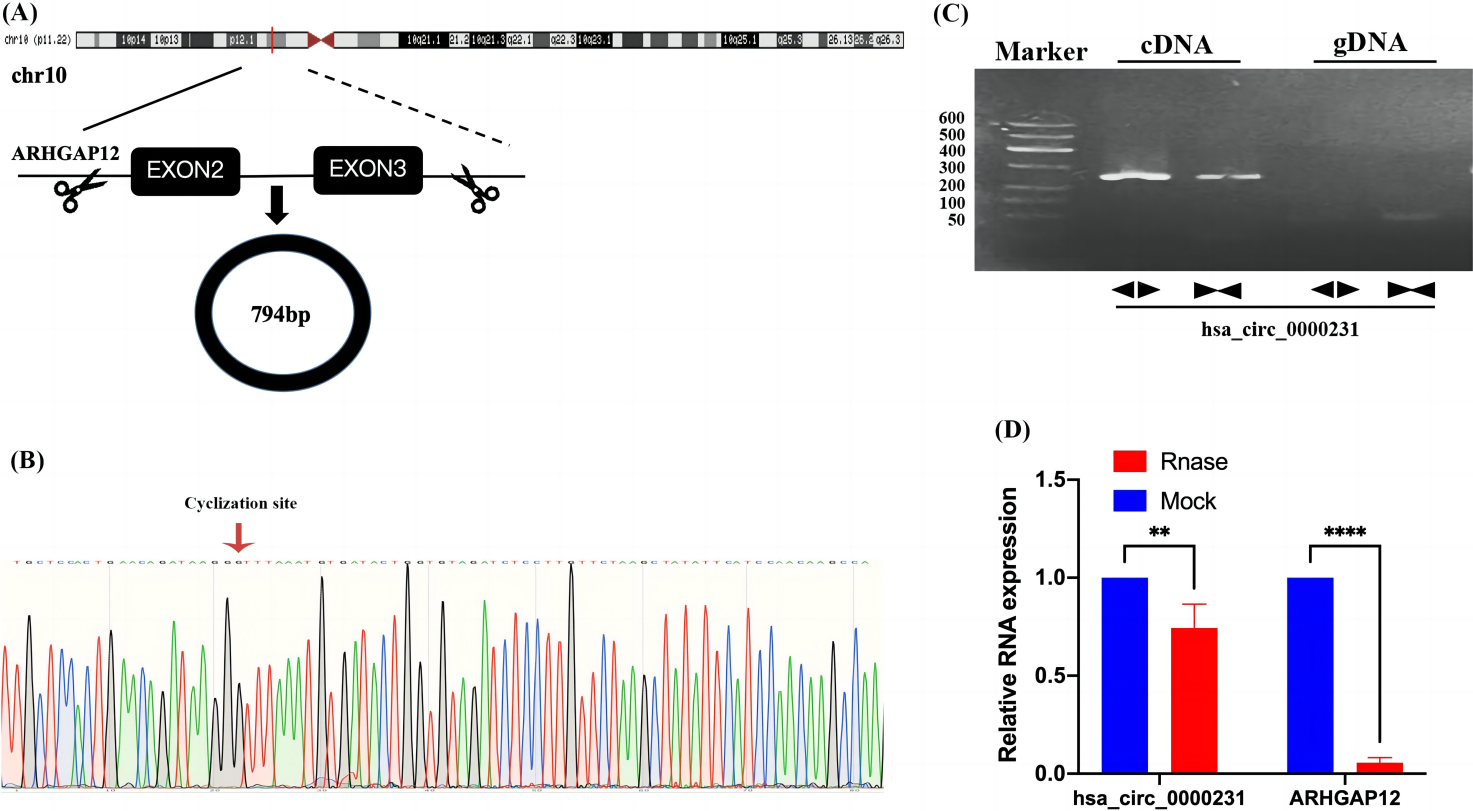
Identification of hsa_circ_0000231 loop structure. (A) Schematic diagram of hsa_circ_0000231 loci and cyclic structure in the genome. (B) The cyclization site of circ_0000231 was identified by Sanger sequencing. (C) RNase R digestion assay to confirm the stability of hsa_circ_00002311. (D) Agarose gel electrophoresis confirmed the cyclic structure of circ_0000231. *p < .05; **p < .01;^****^ *p* < .0001.

According to the circBase database (http://www.circbase.org/), the hsa_circ_0000231 gene on chromosome 10 is the source of hsa_circ_0000231. Exon 2 and exon 3 post‐splicing make up this gene (Figure 1A). The amplification product's cyclization site was verified by Sanger sequencing to be compatible with the cyclization site reported by the CircBase database (Figure 1B). Based on the complementary DNA (cDNA) and genomic DNA (gDNA) of cells, polymerization primers and divergent primers were created. The findings revealed that hsa_circ_0000231 could only be amplified in cDNA (Figure 1C). Additionally, the loop topology of hsa_circ_0000231 prevented Rnase R from quickly degrading, while the expression level of linear hsa_circ_0000231 decreased after degradation, Figure 1D.

### 3.2 Methodological evaluation of hsa_circ_0000231

qRT‐PCR was used to determine the expression of hsa_circ_0000231 in serum, and the findings demonstrated excellent expression in a single peak specificity (Figure SA). Agarose gel electrophoresis was used to validate the qRT‐PCR products. The target segment was amplified by hsa_circ_0000231 to 71 bp and by 18S to 151 bp, and agarose gel electrophoresis findings revealed the predicted size (Figure SB). This demonstrates the method's excellent accuracy and specificity. The linear ranges of hsa_circ_0000231 and 18s rRNA were detected by qRT‐PCR, and the standard curves were displayed. The mixed serum's cDNA was diluted at a 10‐fold ratio. The outcomes revealed that the 18S rRNA standard curve was *Y* = −3.670 × *X* + 8.994, *R* ^2^ = 0.9834, suggesting high linearity, while the standard curve for hsa_circ_0000231 was *Y* = −3.684 × *X* + 22.50, *R* ^2^ = 0.9985, Figure SC,D. The mixed serum was repeatedly freeze–thawed at −80°C and room temperature for 0, 1, 3, 5, and 7 times to extract and detect the expression of hsa_circ_0000231 (*p* < .0001); on the other hand, the mixed serum was left at room temperature for 0, 6, 12, 18, and 24 h to extract and detect the expression of hsa_circ_0000231 (*p* < .0001), and the experiment was repeated three times, and the results showed that the expression of hsa_circ_0000231 was stable (Figure SE,F).

Twenty serum samples were separated into 20 aliquots and mixed at random. The expression levels of hsa_circ_0000231 and 18S were measured in 10 samples from the same batch, and intra‐batch CV was determined based on Ct values. For the remaining 10 samples, one sample was extracted and examined daily, and inter‐batch CV was calculated based on Ct values. Results indicated that the intra‐batch CV and inter‐batch CV of hsa_circ_0000231 and 18S were less than 5%, suggesting high accuracy (Table 1).

**TABLE 1:** The intra‐assay coefficient of variation and the inter‐ assay coefficient of variation of hsa_Circ_0000231.

		hsa_circ_0000231	18s
Intra‐assay	Mean ± SD	31.885 ± 0.650	15.642 ± 0.282
	CV (%)	2.04 (0.650/31.885)	1.81 (0.282/15.642)
Inter‐assay	Mean ± SD	31.905 ± 0.916	15.588 ± 0.265
	CV (%)	2.87 (0.916/31.905)	1.70 (0.265/15.588)

### 3.3 Down‐regulation of hsa_circ_0000231 expression in gastric cancer

**FIGURE 2:**
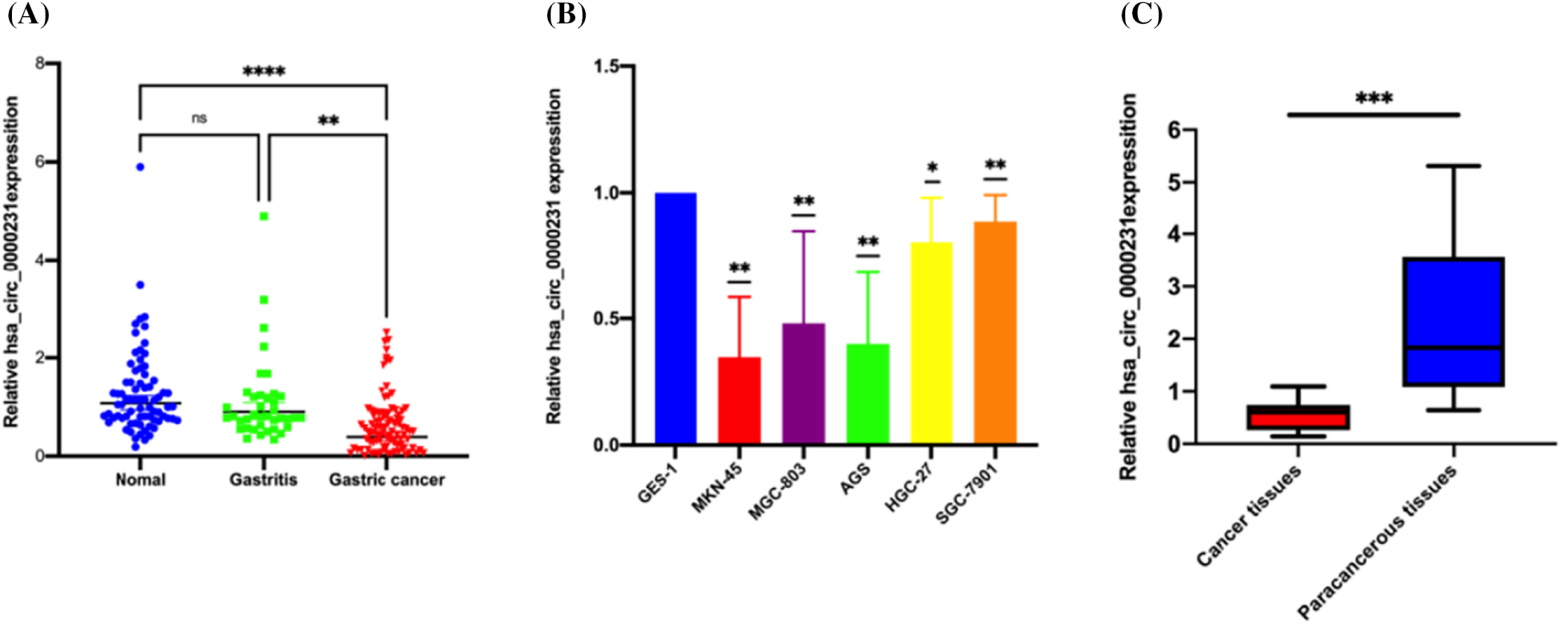
Detection of hsa_circ_0000231 expression. (A) Differential expression of hsa_circ_0000231 in serum. (B) Differential expression of hsa_circ_0000231 in gastric cancer cells. (C) Differential expression of hsa_circ_0000231 in tissues. *p < .05; **p < .01; ***p < .001; ****p < .0001, ns indicates *p* > .05.

The expression levels of hsa_circ_0000231 in the serum of 96 GC patients, 39 gastritis patients, and 74 healthy control patients were investigated by qRT‐PCR. The results revealed that GC patients had considerably lower expression levels than healthy persons (*p* < .0001) and the gastritis patients' group (*p* < .01, Figure 2A). Next, investigation of expression in human gastric mucosal cells (GES‐1) and GC cell lines (HGC‐27, MKN‐45, MGC‐803, AGS) revealed that hsa_circ_0000231 expressions were considerably decreased in cells in line with that in serum, Figure 2B. Further analysis showed that the expression level of hsa_circ_0000231 in GC tissues was significantly lower than that in adjacent tissues, Figure 2C.

### 3.4 hsa_circ_0000231 correlation with clinicopathological parameters

GC patients' clinical and pathological data were gathered, and groups were created based on factors such as gender, age, tumor size, degree of differentiation, TNM stage, lymph node metastasis, and the presence or absence of neurovascular invasion (Table 2). The findings revealed no statistically significant association between gender, age, tumor size, or degree of differentiation (*p* > .05), while low expression of hsa_circ_0000231 was associated with TNM stage (*p* < .05), lymph node metastasis (*p* < .05), and neurovascular invasion in GC patients (*p* < .05).

**TABLE 2:** Relationship between serum hsa_Circ_0000231 expression and clinicopathological parameters in gastric cancer patient.

Characteristic	*n*	hsa_circ_0000231		Pearson *χ* ^2^	*p* value
		Low	High		
Gender					
Male	63	28	35	2.263	.133
Female	33	20	13		
Age (year)					
<70	51	30	21	3.388	.066
≥70	45	18	27		
Size (cm)					
<5	66	31	35	0.776	0.378
≥5	30	17	13		
Differentiation grade					
Poor	39	24	15	3.498	.061
Moderate	57	24	33		
T stage					
T1 and T2	41	15	26	5.151	.023*
T3 and T4	55	33	22		
Lymphatic metastasis					
Yes	54	32	22	4.233	.04*
No	42	16	26		
TNM stage					
I and II	57	23	34	5.225	.022*
III and IV	39	25	14		
Neural/vascular differentiation					
Yes	55	36	19	12.303	<.0001^****^
No	41	12	29		

*Note*: Pearson *χ* ^2^ test was used in statistical analysis.

* *p* < .05;

^****^ *p* < .0001.

### 3.5 Diagnostic value of hsa_circ_0000231 and the combined diagnostic efficacy with CEA, CA19‐9, and CA72‐4

**FIGURE 3:**
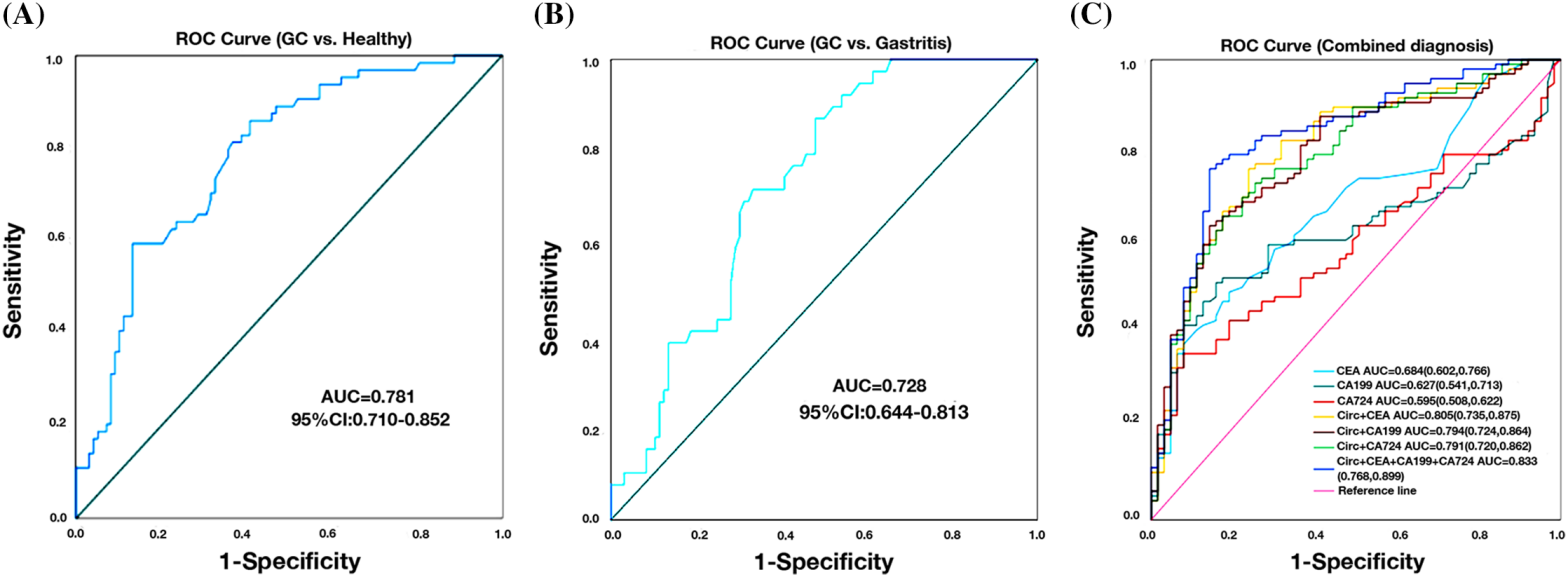
Potential of hsa_circ_0000231 as a diagnostic biomarker. (A) receiver operating characteristic (ROC) curve analysis of plasma hsa_circ_0000231 to identify primary gastric cancer (GC) patients from healthy donors (AUC = 0.781). (B) ROC curve analysis of plasma hsa_circ_0000231 for the identification of patients with primary gastric cancer and patients with gastritis (AUC = 0.728). (C) CEA: AUC = 0.684, CA19‐9: AUC = 0.627, CA72‐4: AUC = 0.595 and combined diagnostic efficacy: AUC = 0.833.

Then, as a possible biomarker for GC, we further investigated the characteristics of hsa_circ_0000231. ROC curves and AUC of ROC curves were run on data from 96 people with gastric cancer and 74 healthy people to see if serum hsa_circ_0000231 could be used as a biomarker for diagnosing gastric cancer. The outcomes demonstrated that serum hsa_circ_0000231 had an AUC of 0.781 (95% CI: 0.716–0.852) and was useful in differentiating patients with primary GC from healthy individuals (Figure 3A). Adjuvant screening signs for gastrointestinal tract cancers include CEA, CA19‐9, and CA72‐4. The diagnostic performance of CEA (AUC = 0.684, 95% CI: 0.602–0.766), CA19‐9 (AUC = 0.627, 95% CI: 0.541–0.713), and CA72‐4 (AUC = 0.595, 95% CI: 0.508–0.622) was worse to that of hsa_circ_0000231. The combination of serum hsa_circ_0000231, CEA, CA19‐9, and CA72‐4 was superior to any of the biomarkers examined alone in identifying GC patients (AUC = 0.833, 95% CI: 0.768–0.899), Figure 3B. Contrary to CEA (33.67%), CA19‐9 (40.82%), and CA72‐4 (32.65%), The sensitivity and specificity of hsa_circ_0000231 were 86.73%, 57.81%, respectively. Hsa_circ_0000231, CEA, and CA19‐9 together increased diagnostic sensitivity (97.96%), Table 3. The ability of hsa_circ_0000231 to differentiate between patients with primary gastric cancer and gastritis was equally good (AUC = 0.728, 95% CI: 0.644–0.813) (Figure 3C).

**TABLE 3:** Evaluation of the diagnostic value in patients with gastric cancer versus healthy physical examiners.

	SEN (%)	SPE (%)	ACCU (%)	PPV (%)	NPV (%)
Hsa_Circ_0000231	86.73 (85/98)	57.81 (37/64)	75.31 (122/162)	75.89 (85/112)	74.00 (37/50)
CEA	33.67 (33/98)	92.19 (59/64)	56.79 (92/162)	86.84 (33/38)	45.74 (59/129)
CA19‐9	40.82 (40/98)	90.63 (58/64)	60.49 (98/162)	86.96 (40/46)	50.00 (58/116)
CA72‐4	32.65 (32/98)	92.19 (59/64)	56.17 (92/162)	86.49 (32/37)	43.70 (59/135)
Hsa_Circ_0000231 + CEA	91.84 (90/98)	53.13 (34/64)	76.54 (124/162	75.00 (90/120	80.95 (34/42)
Hsa_Circ_0000231 + CA19‐9	94.90 (93/98)	53.13 (34/64)	78.40 (127/162)	75.61 (93/123)	87.18 (34/39)
Hsa_Circ_0000231 + CA72‐4	92.86 (91/98)	54.69 (35/64)	77.78 (126/162)	75.83 (91/120)	83.33 (35/42)
Hsa_Circ_0000231 + CEA + CA19‐9 + CA72‐4	97.96 (96/98)	51.56 (33/64)	79.63 (129/162)	75.59 (96/127)	94.29 (33/35)

Abbreviations: ACCU, overall precision; NPV, negative predictive value; PPV, positive predictive value; SEN, sensitivity; SPE, specificity.

### 3.6 The role of serum hsa_circ_0000231 in tumor dynamic monitoring of gastric cancer patients

**FIGURE 4:**
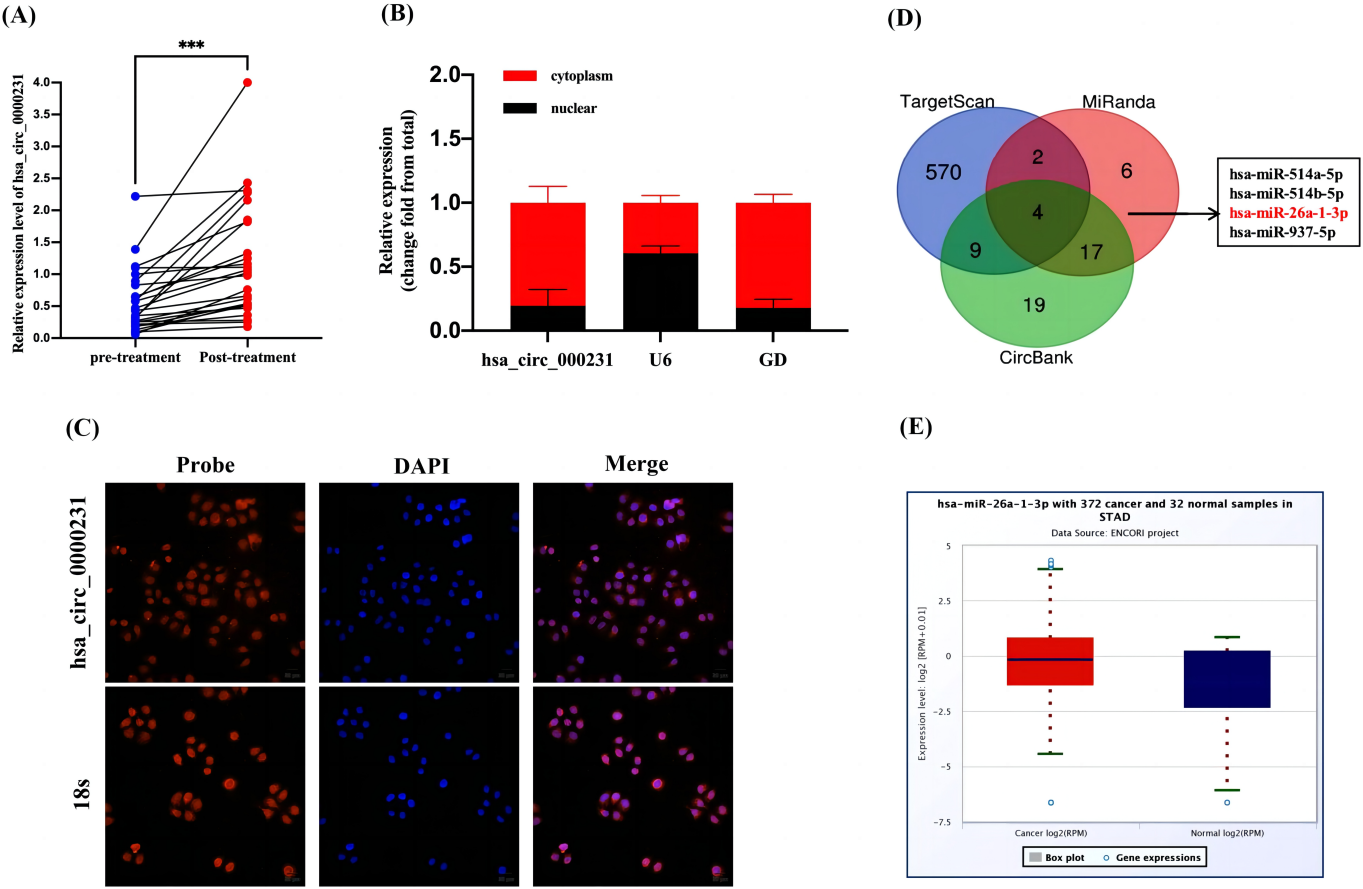
Dynamic monitoring of hsa_circ_0000231 and prediction of downstream regulatory network. (A) Changes in hsa_circ_0000231 expression before and after treatment (*p* = .001, *n* = 26). (B) Nuclear plasma isolation assay to determine the binding site of hsa_circ_0000231 in GC cell line MKN‐45. (C) Fluorescence in situ hybridization (FISH) analysis of the cellular distribution of hsa_circ_0000231 and 18s. (D) Bioinformatics analysis was used to predict and screen possible miRNA candidates. (E) miRNA with expression levels in gastric cancer predicted by starbase.***p < .001.

To verify the dynamic relationship between plasma hsa_circ_0000231 expression and tumor progression, we compared the preoperative and postoperative paired serum hsa_circ_0000231 expression levels in 26 GC patients. The expression level of hsa_circ_0000231 was significantly lower in preoperative than in postoperative (*p* = .001), which offers the possibility of detecting tumor dynamics and prognosis (Figure 4A).

### 3.7 Forecast hsa_circ_0000231's downstream regulatory network in GC

It was found that miRNAs can act as sponges for circRNAs involved in the construction of competitive endogenous RNAs regulating tumor progression.^21^, ^22^ Nucleoplasmic separation experiments and FISH showed that hsa_circ_0000231 was slightly more abundant in the cytoplasm than in the nucleus, indicating that it was mainly located in the cytoplasm (Figure 4B,C), which suggests that it may have a post‐transcriptional regulatory function. Next, we predicted the downstream miRNAs by bioinformatics databases (CircBank, http://www.circbank.cn/; Miranda, http://www.microrna.org/; TargetScan, https://www.targetscan.org/vert_80/), and obtained hsa‐miR‐514a‐5p, hsa‐miR‐514b‐5p, hsa‐miR‐26a‐1‐3p, hsa‐miR‐937‐5p (Figure 4D). Then Searching for these miRNAs in the ENCORI (https://rnasysu.com/encori/) database, only miR‐26a‐1‐3p expression was down‐regulated among them, which provided a new direction for subsequent studies of hsa_circ_0000231 (Figure 4E).

## 4 DISCUSSION

Gastric cancer is a malignant tumor that arises from the stomach mucosa epithelium and has a poor prognosis and a high death rate.^23^, ^24^ Chemotherapy still poses a serious hazard to human health despite being used increasingly often in recent years to treat advanced stomach cancer. Currently, invasive tissue biopsy and cytological examination are still required for the early identification of stomach cancer, but the liquid biopsy, a non‐invasive tumor diagnostic method, is slowly coming into scientific view.^25^, ^26^ In clinical practice, the biomarkers CEA and CA19‐9 are frequently employed for adjuvant diagnosis of gastrointestinal cancers.^27^ Nevertheless, the early identification and prognosis of many malignancies are not restricted by the low sensitivity and specificity of these tests.^28^, ^29^ Therefore, the search for effective and sensitive biomarkers is crucial.

Circular RNAs are a special class of non‐coding RNAs that are structurally characterized by closed loops and do not possess conventional 5′ and 3′ ends.^30^ Due to the rapid development of high‐throughput transcriptome analysis tools, thousands of circRNAs have been identified through the detection of body fluids such as plasma, serum, exosomes, and urine.^31^ CircRNAs, as a special class of RNA molecules, are endowed with distinctive biological functions due to their unique loop structure. The emergence of more and more data invariably reveals a striking fact: circRNAs not only play a crucial role in the onset and progression of many diseases, but also have a close association with tumor growth and proliferation.^32^ These research results not only highlight the great potential of circRNAs as novel biomarkers for human tumors, but also demonstrate their broad application prospects in the field of therapeutic targets. For example, hsa_circ_0007813 was up‐regulated in bladder cancer, and its high expression was associated with increased tumor size, primary tumor T‐stage, and pathological grading.^33^ Xi et al. found that CircBCAR3 promoted the proliferation, migration, and invasion of esophageal cancer cells.^34^ Circ_0011292 could be partially regulated through the regulation of Circ _0011292/miR‐433‐3p/CHEK1 axis to accelerate PTX resistance and cellular malignant progression in NSCLC cells.^35^

In this study, by using AGE, Sanger sequencing, and qRT‐PCR in this investigation, the single‐peak specificity molecule hsa_circ_0000231 was discovered. After that, the assay was assessed for intra‐ and inter‐batch variance. CV values were less than 5%, which showed good accuracy. The linear range and stability of the specimens were verified by several freeze–thaw experiments at room temperature. The serum of 96 GC patients and 15 samples of GC tissue showed that the expression level of hsa_circ_0000231 was much lower than that of healthy donors. This suggests that it may be a gene that stops cancer from growing. Based on the clinicopathological data of 96 patients, it was found that low expression of hsa_circ_0000231 in serum was related to TNM stage, lymph node metastasis, and nerve/vasculature invasion, it indicates that hsa_circ_0000231 may be a related molecule in the progression and metastasis of GC. According to the findings of ROC curve analysis, hsa_circ_0000231 had a formidable diagnostic value and could clearly separate primary gastric cancer patients from the healthy group and patients with gastritis. hsa_circ_0000231, CEA, CA19‐9, and CA72‐4 together have a sensitivity of 97.96% and a specificity of 51.56% for the diagnosis of GC. Additionally, postoperative patients serum showed elevated levels of hsa_circ_0000231 expression, which raises the possibility of a real‐time monitoring role.

MiRNAs can build a sizable regulatory network in the transcriptome, greatly expanding the functional gene data in the human genome and playing a crucial role in pathological conditions such as cancer.^36^, ^37^, ^38^, ^39^ CircRNAs can act as “competing endogenous RNAs” (ceRNAs) interacting with miRNAs to influence tumor progression. In this study, nucleoplasmic isolation and FISH assays demonstrated that hsa_circ_0000231, which is mainly distributed in the cytoplasm, can act as a miRNA sponge and participate in regulating the course of gastric cancer.

## 5 CONCLUSION

In conclusion, our study revealed for the first time that the expression level of hsa_circ_0000231 was down‐regulated in GC tissues, cells and serum and its expression level increased after gastric cancer surgery, suggesting that it can be used for dynamic monitoring of gastric cancer patients. In addition, the ROC curve also demonstrated the combined diagnostic ability of hsa_circ_0000231 with CEA, CA19‐9 and CA72‐4. Based on our results, hsa_circ_0000231 has the potential to be a biomarker for GC diagnosis and prognostic monitoring, but its specific function and mechanism need to be further investigated. Furthermore, it is pertinent to note that the study possesses certain constraints, including a comparatively limited sample size, an absence of extended follow‐up period, and the necessity for more uniform protocols tailored for clinical utilization. But the search for specific and sensitive biomarkers is undoubtedly the way forward for tumor diagnosis and prognosis. We shall await the speedy advancement of non‐coding RNA with patience and a keen eye.

## AUTHOR CONTRIBUTIONS

**Ronghua Fang:** Conceptualization (equal); data curation (equal); formal analysis (equal); investigation (equal); methodology (equal); project administration (equal); software (equal); validation (equal); visualization (equal); writing – original draft (equal); writing – review and editing (equal). **Wentao Yuan:** Conceptualization (equal); data curation (equal); formal analysis (equal); investigation (equal); methodology (equal); project administration (equal); software (equal); validation (equal); visualization (equal); writing – original draft (equal); writing – review and editing (equal). **Chunyan Mao:** Data curation (equal); formal analysis (equal); investigation (equal); methodology (equal); software (equal); validation (equal); visualization (equal); writing – original draft (equal); writing – review and editing (equal). **Jing Cao:** Data curation (equal); formal analysis (equal); investigation (equal); methodology (equal); software (equal); writing – original draft (equal); writing – review and editing (equal). **Hongmei Chen:** Data curation (equal); formal analysis (equal); investigation (equal); methodology (equal); writing – original draft (equal); writing – review and editing (equal). **Xiuying Shi:** Conceptualization (equal); supervision (equal); writing – original draft (equal); writing – review and editing (equal). **Hui Cong:** Conceptualization (equal); funding acquisition (equal); resources (equal); supervision (equal); writing – original draft (equal); writing – review and editing (equal).

## CONFLICT OF INTEREST STATEMENT

The authors have stated explicitly that there are no conflicts of interest in connection with this article.

## ETHICS STATEMENT

The research protocol was approved by the medical ethics committee of Affiliated Hospital of Nantong University (approval number: 2021‐L033) and conducted in accordance with the Declaration of Helsinki. The need for informed consent was waived by the medical ethics committee of Affiliated Hospital of Nantong University.

## Supporting information


**FIGURE S** Hsa_circ_0000231 methodological evaluation. A. Hsa_circ_0000231 and 18s amplification curve with single peak specificity. B. Agarose gel electrophoresis to verify the correctness of hsa_circ_0000231 and 18s. C‐D. Hsa_circ_0000231 and 18s linear range evaluation. E‐F. Hsa_circ_0000231 and 18s stability evaluation.

## Data Availability

Data are available from the corresponding author upon request.
