# Supplementary material for: Human circular RNA hsa_circ_0000231 clinical diagnostic effectiveness as a new tumor marker in gastric cancer
Source: Cancer Rep (Hoboken). 2024 May 4;7(5):e2081. doi: 10.1002/cnr2.2081 (PMC11069127; doi:10.1002/cnr2.2081)
Supplement: Supplementary file 1 — FIGURE S Hsa_circ_0000231 methodological evaluation. A. Hsa_circ_0000231 and 18s amplification curve with single peak specificity. B. Agarose gel electrophoresis to verify the correctness of hsa_circ_0000231 and 18s. C‐D. Hsa_circ_0000231 and 18s linear range evaluation. E‐F. Hsa_circ_0000231 and 18s stability evaluation. [file CNR2-7-e2081-s001.docx]

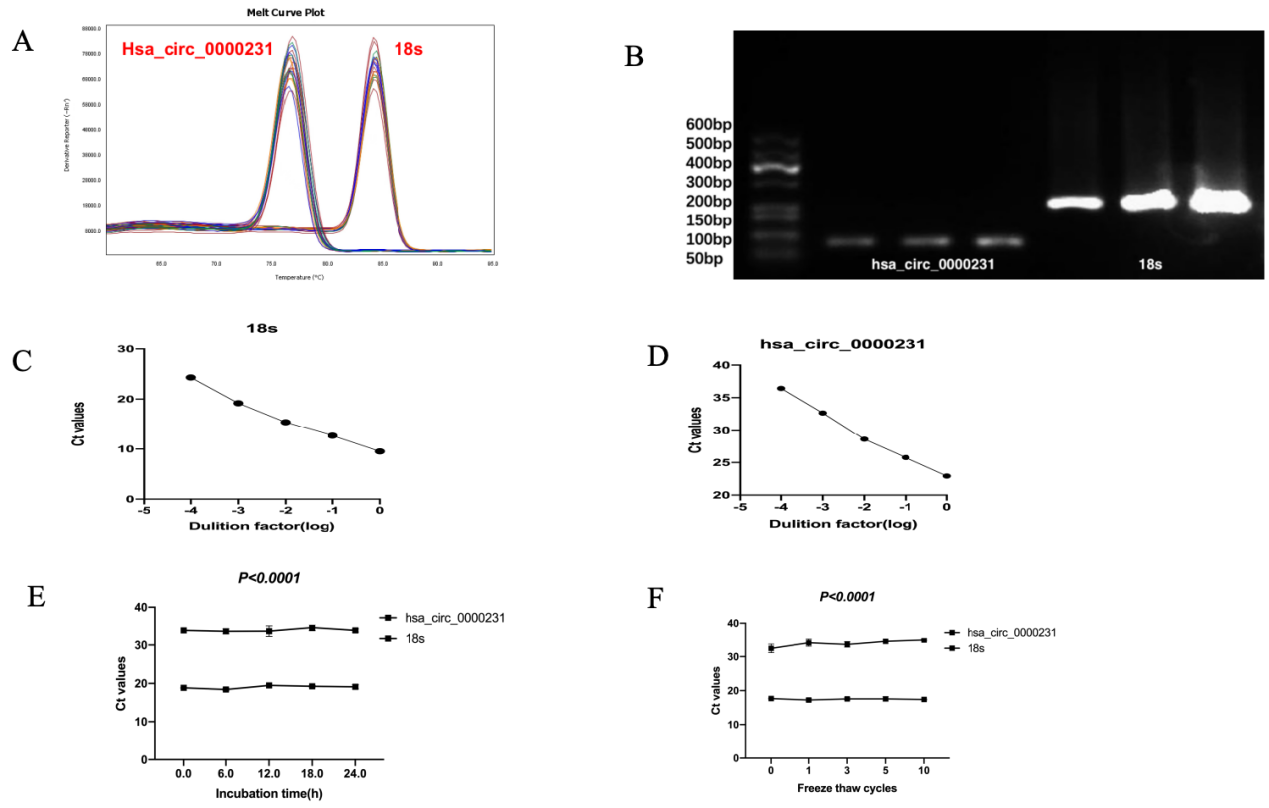


FIGURE S Hsa_circ_0000231 methodological evaluation. A. Hsa_circ_0000231 and 18s amplification curve with single peak specificity. B. Agarose gel electrophoresis to verify the correctness of hsa_circ_0000231 and 18s. C-D. Hsa_circ_0000231 and 18s linear range evaluation. E-F. Hsa_circ_0000231 and 18s stability evaluation.
